# What Motivates People With (Pre)Diabetes to Move? Testing Self-Determination Theory in Rural Uganda

**DOI:** 10.3389/fpsyg.2020.00404

**Published:** 2020-03-24

**Authors:** Jeroen De Man, Edwin Wouters, Pilvikki Absetz, Meena Daivadanam, Gloria Naggayi, Francis Xavier Kasujja, Roy Remmen, David Guwatudde, Josefien Van Olmen

**Affiliations:** ^1^Centre for General Practice, Department of Primary and Interdisciplinary Care, University of Antwerp, Antwerp, Belgium; ^2^Centre for Population, Family and Health, Department of Sociology, University of Antwerp, Antwerp, Belgium; ^3^Collaborative Care Systems Finland, Helsinki, Finland; ^4^Institute of Public Health and Clinical Nutrition, University of Eastern Finland, Kuopio, Finland; ^5^Department of Food Studies, Nutrition and Dietetics, Uppsala University, Uppsala, Sweden; ^6^Health Systems and Policy Research Group, Department of Global Public Health, Karolinska Institutet, Stockholm, Sweden; ^7^International Maternal and Child Health Division, Department of Women’s and Children’s Health, Uppsala University, Uppsala, Sweden; ^8^Department of Epidemiology and Biostatistics, School of Public Health, College of Health Sciences, Makerere University, Kampala, Uganda; ^9^Department of Public Health, Institute of Tropical Medicine Antwerp, Antwerp, Belgium

**Keywords:** type 2 diabetes, physical activity, self-determination theory, sub-saharan Africa, Uganda, psychological needs theory, autonomous motivation, controlled motivation

## Abstract

**Introduction:**

Sub-Saharan Africa is experiencing a rapid growth of type 2 diabetes (T2D) and its related burden. Regular physical activity (PA) is a successful prevention strategy but is challenging to maintain. Self-determination theory (SDT) posits that more autonomous forms of motivation are associated with more sustainable behavior change. Evidence to support this claim is lacking in sub-Saharan Africa. This study aims to explore the relationships between latent constructs of autonomous and controlled motivation, perceived competence, perceived relatedness, PA behavior, and glycemic biomarkers.

**Methods:**

Structural equation modeling was applied to cross-sectional data from a rural Ugandan population (*N* = 712, pre-diabetes = 329, diabetes = 383). Outcome measures included self-reported moderate and vigorous PA, pedometer counts, and fasting plasma glucose (FPG) and glycated hemoglobin (HbA1C).

**Results:**

Our findings support SDT, but also suggest that different types of motivation regulate different domains and intensities of PA. Higher frequency of vigorous PA – which was linked to a lower HbA1C and FPG – was predicted by autonomous motivation (β = 0.24) but not by controlled motivation (β = −0.05). However, we found no association with moderate PA frequency nor with pedometer counts. Perceived competence and perceived relatedness predicted autonomous motivation. Autonomous motivation functioned as a mediator between those needs and PA behavior.

**Conclusion:**

This is the first study providing evidence for a SDT model explaining PA among people at risk of, or living with, T2D in a rural sub-Saharan African setting. Our findings suggest that individuals who experience genuine support from friends or family and who feel competent in doing vigorous PA can become motivated through identification of health benefits of PA as their own goals. This type of motivation resulted in a higher frequency of vigorous PA and better glycemic biomarkers. On the other hand, people who felt more motivated through pressure from others or through feelings of guilt or shame were not more engaged in PA.

**Clinical Trial Registration:**

ISRCTN 11913581. Registered January 10, 2017.

## Introduction

Diabetes is among the world’s ten biggest killers and is a leading cause of disability (International Diabetes Federation, 2017). T2D is by far the most common form of diabetes and its global prevalence is predicted to grow from 424.9 million in 2017 to 628.6 million in 2040 (International Diabetes Federation, 2017). The sub-Saharan region will be an important contributor to this rapid growth which risks reversing some of the recent health gains in this region. The massive burden of diabetes is estimated to drain national healthcare budgets and to overwhelm the often fragile and underfunded health systems in Sub-Saharan Africa (Atun et al., 2017; International Diabetes Federation, 2017). Costs associated with diabetes could more than double in Sub-Saharan Africa, reaching up to United States $59.3 billion per year by 2030 (Atun et al., 2017). Furthermore, T2D is known to strike people at their most productive age and cripple their personal finances which will reduce their productivity, slow economic growth, and increase poverty rates (International Diabetes Federation, 2017).

Important drivers behind the African diabetes pandemic are rapid societal transitions resulting in more sedentary work practices, changing lifestyle habits, and an increase in wealth and aging populations (Atun et al., 2017). This suggests that primary prevention through lifestyle modification will be pivotal in curbing the diabetes pandemic (Steyn and Damasceno, 2006). Engaging in regular PA, for example, has been shown to substantially reduce the risk of diabetes onset and its complications (Helmrich et al., 1991). A beneficial effect of PA has also been shown for other non-communicable diseases, including cardiovascular and mental health-related diseases (Reiner et al., 2013).

A recent systematic review on self-management of T2D in Sub-Saharan Africa found out that, generally, people living with T2D are aware of the importance of aerobic exercises (Stephani et al., 2018). However, reported adherence rates to exercise plans were low (between 29 and 46%). Study populations showed only moderate adherence to other self-management activities such as adherence to diet plans and medication schemes and self-monitoring of blood glucose (Stephani et al., 2018). Despite people being aware of the potential benefits, sustainable behavior change such as engaging in regular PA turns out to be challenging and has been suggested to depend on a supportive physical and social environment (De Man et al., 2019). However, the mechanisms through which people’s social environment influences behavior change are poorly understood in sub-Saharan African settings (Sallis et al., 2016; Stephani et al., 2018). To design and implement effective behavior change interventions at the public health level, an evidence-based framework is needed, especially in those countries that will experience the biggest growth in lifestyle-related diseases.

Self-Determination Theory provides a potential explanatory theoretical framework that connects people’s social environment to motivation and behavior change. SDT has received extensive scientific attention in predicting healthy behaviors, such as PA (Teixeira et al., 2012). Central to SDT is the differentiation between controlled and autonomous forms of motivation based on their degree of self-determination (Deci and Ryan, 2000). Controlled motivation can be triggered by sources external to the actual behavior, such as incentives or perceived approval from others (i.e. external regulation), or by internal pressures, such as self-worth, pride, or threats of guilt and shame (i.e. introjected regulation) (Deci and Ryan, 2000). Individuals moved by controlled forms of motivation are likely to quickly lose interest once the external factor is removed (Deci and Ryan, 2000).

Autonomous forms of motivation emanate from within oneself or from abiding values and lead to enhanced performance and persistence in comparison with controlled motivation (Deci and Ryan, 2000). Identified regulation is a type of autonomous motivation that is particularly relevant to a specific health behavior like PA (Teixeira et al., 2012). Identified regulation of PA is triggered by the individual associating PA with their personal goals or values, such as “being healthy”. Through this process of identification, individuals endorse their own actions, which is a behavior that has been associated with a higher commitment and performance level (Deci and Ryan, 2000). Identified regulation has been estimated to be a more consistent predictor of regular PA than other forms of autonomous motivation, such as pure intrinsic regulation which is triggered by pleasure derived from the activity itself (Levesque et al., 2007; Teixeira et al., 2012). A plausible explanation would be that PA often relates to more mundane or repetitive actions (Teixeira et al., 2012).

The basic psychological needs theory, which is part of SDT, posits that one’s social context plays a key role in how one is motivated. The theory claims that social contexts that support the individual’s perceived competence, autonomy, and relatedness (i.e. the three basic psychological needs), will foster more autonomous types of motivation and thus contribute to persistence in one’s actions (Deci and Ryan, 2000). Perceived competence corresponds to the sense of efficacy one has with respect to both internal and external environments (Ryan et al., 2008). Perceived autonomy refers to a sense of choice and volition in the regulation of one’s behavior. Perceived relatedness corresponds to the sense of being supported by significant others in one’s actions. According to basic psychological needs theory, interventions aiming for a sustainable change in people’s behavior should primarily focus on conditions that enhance the individual’s basic psychological needs. In contrast, the individual’s context may also thwart their experience of basic needs which will result in less autonomous forms of motivation and, according to basic psychological needs theory, a lower performance. Several studies in western countries have provided evidence on the role of SDT in explaining PA engagement among people living with T2D. The majority of those studies found a positive association between autonomous forms of motivation and PA (Sweet et al., 2009; Fortier et al., 2012; Miquelon and Castonguay, 2016; Gourlan et al., 2016; Koponen et al., 2017), some claiming that it is the most important factor in compliance with PA recommendations (Juul et al., 2018). Studies also support the SDT process model with the basic psychological needs associated with higher PA engagement and better clinical outcomes (Williams et al., 2004; Halvari et al., 2017). This evidence is claimed to be cross-culturally and universally applicable (Deci and Ryan, 2000). However, evidence on the psychosocial mechanism is very limited in sub-Saharan African settings (Sallis et al., 2016; Stephani et al., 2018). And to the best of our knowledge, no studies have addressed the relationship between SDT constructs and PA among people at risk of, or living with, non-communicable diseases in the sub-Saharan subcontinent. Moreover, our study connects the SDT constructs – through PA behavior – with HbA1C and FPG, which are important clinical parameters in the prevention and management of T2D.

Previous studies that have investigated SDT and PA in western contexts also had several limitations: (1) a majority of studies have been limited to bivariate analyses between motivational constructs and PA, whereas only a few found evidence for a mediation effect on autonomous motivation (Weman-Josefsson et al., 2015); (2) studies have often been limited to one specific, or an aggregated outcome of, PA, whereas the role of SDT has been suggested to depend on the type and intensity of the activity (Teixeira et al., 2012); (3) existing studies have often relied on self-reported outcomes which can strongly differ from more objective measures obtained by pedometers or accelerometers (Teixeira et al., 2012; Rhodes et al., 2017); and (4) earlier studies typically used a unidimensional index of the degree of self-determination (Teixeira et al., 2012), ignoring the multi-dimensionality of the SDT model (Chemolli and Gagné, 2014).

This study uses structural equation modeling to test the validity of the SDT framework with regards to moderate PA, vigorous PA, and counted steps by a pedometer. The study focuses on people newly diagnosed with, or at high risk of, T2D because we believe that understanding ongoing motivational dynamics is crucial for the promotion of dietary behavior change. The study population from rural Uganda is socio-economically disadvantaged and has a low education level (De Man et al., 2019). This population is similar to populations in other Sub-Saharan African countries in terms of demographic and socio-economic aspects. Hence, we deem the results representative for other adult populations including populations at risk of, or living with, NCDs such as cardiovascular or respiratory conditions.

**FIGURE 1:**
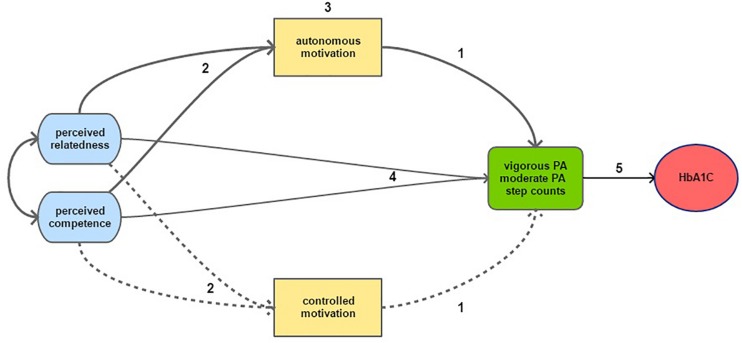
Graphical representation of the hypotheses tested in this study, including 2 of the 3 psychological needs: perceived competence and perceived relatedness. Solid arrows represent hypothesized positive effects and the dotted arrows represents hypothesized non-associations or negative effects. Numbers refer to the hypothesis discussed in the text.

We hypothesized the following relationships (see numbers mentioned in Figure 1): (1) a positive effect of autonomous motivation and no effect of controlled motivation on PA; (2) according to the basic psychological needs theory, we hypothesized a positive effect of perceived relatedness and perceived competence on autonomous motivation and a no effect of those needs on controlled motivation. Whereas perceived autonomy is one of the three psychological needs, no appropriate measure was available at the start of our study and we focused our attention on the two other needs; (3) we hypothesized that autonomous motivation would at least partially mediate the effect of perceived relatedness and perceived competence; (4) a positive total effect of perceived relatedness and perceived competence on PA outcomes; and (5) a positive effect of PA on HbA1C and FPG. The correlation allowed between perceived relatedness and competence is in line with SDT models in other domains, such as work (Eriksson and Boman, 2018).

## Materials and Methods

### Study Design and Procedures

This study is part of SMART2D, a cluster-randomized adaptive implementation trial aimed at improving self-management among people at risk of, or living with, T2D (Guwatudde et al., 2018). The present study is part of the validation of a theory-driven framework that guided the implementation of the SMART2D intervention trial (De Man et al., 2019). The study analyzed cross-sectional baseline data collected from Iganga and Mayuge, two rural districts in eastern Uganda.

### Study Participants and Recruitment

Participants were residents of geographically determined catchment areas of nine primary healthcare facilities, aged 30–75 years, with a positive confirmatory test for prediabetes or diabetes, and did not suffer from serious mental disability. More details about the inclusion and exclusion criteria are published elsewhere (Guwatudde et al., 2018).

A total of 794 participants were recruited by trained field research assistants approaching households in the study area clusters. After having given written informed consent, potential participants were screened for eligibility based on the pre-defined inclusion and exclusion criteria. Eligible individuals underwent two FPG tests on two different days. If they met the criteria for prediabetes or diabetes, they were invited to enroll in the study at the primary health center in their catchment area. A third FPG test was performed at the health facility to confirm prediabetes or diabetes. More details about the recruitment process can be found elsewhere (Guwatudde et al., 2018).

### Data Collection

Initially, trained study staff administered a structured questionnaire to obtain, among other data, socio-demographic data and PA measures from eligible participants. In addition, blood samples were collected to test HbA1C and FPG. Participants were re-invited after 7 days to collect the pedometer counts and to respond to a second structured questionnaire with questions on motivational constructs. The questionnaire was split into two parts to minimize participant fatigue. Participants were enrolled between January 2017 through December 2017.

### Missing Data

The motivational constructs questionnaire was not administered to 82 (10,3%) of the 794 participants. This group did not significantly differ in PA outcomes compared to the rest of the participants and was deleted listwise. Among the remaining 712 participants, the percentage of missing data was 10.1 for the pedometer counts and varied between 0.0 and 2.7 for the other variables. Multivariate imputation by chained equations with predictive mean matching was used to handle the missing data under a missing at random assumption. Rubin’s rules were used to pool point and SE estimates across 20 imputed data sets. The procedure was done using the “Mice” package in R.

### Measures

The SDT concept of *perceived competence* was measured through barrier self-efficacy (or self-regulatory efficacy) which corresponds to the perceived capability to maintain PA in given various conditions or impediments (i.e. barriers). This proxy measure was preferred because of its adaptability to different outcomes and (non-western) contexts. Six items were created to assess self-belief in coping with a variety of barriers to physical exercise, as proposed by Schwarzer et al. (2007) and Renner et al. (2012) (see Supplementary File 1). The items retain a common semantic structure: “Do you think you can do X, even if Y (barrier)”, for example: “Do you think you can be physically active even if you think it is not the best weather for doing sports?”. Barriers used in the initial studies were modified to barriers relevant to our study population and relevant to the PA outcomes used in this study. Participants responded to each item on a 5-point Likert-type scale ranging from 1 (strongly disagree) to 5 (strongly agree).

To measure the concept of *perceived relatedness*, we used an adapted and shortened version of the scale for participation and involvement of family members and friends in PA as a proxy measure. This scale was developed by Sallis et al. (1987) and has been used and validated in a variety of contexts. Five items of the initial measure were selected based on their cross-cultural relevance and factor loadings in previous studies (Sallis et al., 1987) (see Supplementary File 1). The questions shared the stem: “How often have people close to you (friends, family, or relatives)?”, followed by items such as “exercised with you,” “encouraged you to exercise,” etc. Possible responses included: “Never,” “less than once a week,” “once a week,” and “more than once a week.” To emphasize the concept of perceived support among the study participants, we introduced the questions with the following statement: *“We want to understand to what extent people close to you (friends, family, or relatives) have helped you to do physical activity.”*

*Autonomous and controlled motivation toward physical exercise* were assessed through the Treatment Self-Regulation Questionnaire (TSRQ) for people with diabetes. The TSRQ has been widely used to test autonomous self-regulation with regards to physical exercise (Teixeira et al., 2012). Validation studies (Levesque et al., 2007) and studies investigating physical exercise among diabetes patients (Williams et al., 1998) report adequate reliability and validity of the questionnaire. Guided by factor loadings identified in a validation study by Levesque et al. (2007), eight items were selected: four measuring autonomous motivation (i.e. identified regulation) and four measuring controlled motivation (i.e. external and introjected regulation). Items belonging to different types of motivation were mixed when presented to participants to minimize acquiescence bias (see Supplementary File 1). Originally, the items were statements which we transformed to questions asking why participants would engage in PA. An example of the autonomous reasons is: “Would you do PA because you feel that you want to take responsibility for your own health?” and an example of the controlled reasons is: “Would you do PA because you would feel guilty or ashamed of yourself if you didn’t?”. Participants responded to each item on a 5-point Likert-type scale ranging from 1 (strongly disagree) to 5 (strongly agree).

Physical activity was measured through: (*1) self-reported frequency of vigorous PA, (2) self-reported frequency of moderate PA, and (3) pedometer counts*. Different measures were used since associations with SDT constructs may depend on the type and intensity of PA (Teixeira et al., 2012). Moreover, pedometer counts were used because self-reported measures are subject to recall and social desirability bias (Rhodes et al., 2017). Pedometer outputs have been shown to closely correlate with the output of accelerometers (Tudor-Locke et al., 2002).

Pedometers, however, do not fully capture the intensity of activities that are not step-based (e.g. digging, bicycling, etc.) (Rhodes et al., 2017). *Frequency of vigorous and moderate* PA were measured through two questions modified from the WHO STEPS survey (World Health Organization, 2017) with answer options 0 to 7. Participants were asked: “On how many of the last 7 days did you do vigorous PA for at least 15 min?” and “On how many of the last 7 days did you do moderate PA for at least 30 min?” Contextual examples of what vigorous or moderate activities could entail were included in the questions (see Supplementary File 1). Yamax Digiwalker SW-200 pedometers were used to count the number of steps taken by each participant. Among the spring-levered pedometers this type has been considered the most accurate and most suitable for research purposes (Tudor-Locke et al., 2011). To avoid bias through variation in the number of weekdays versus weekends, only participants who wore the pedometer for 7 or a multiple of 7 consecutive days were considered. Participants were instructed face-to-face to always (day and night) wear the pedometer around the waist. The accumulated number of steps was then recorded during their next interview. This approach was reported to produce an accurate estimate of the steps taken under free living conditions (Tudor-Locke et al., 2011). Values of <1000 or >25,000 steps/day were set as missing as recommended for data reduction (Tudor-Locke et al., 2011).

To measure HbA1c and FPG, capillary blood samples were taken from the fingertip of each enrolled participant. Measurement of HbA1C was done by a qualified laboratory technician, using a point of care HbA1c Analyzer Cobas b101 (Roche Diagnostics), with the respective test and control reagents. Measurement of FPG was done using Accucheck^®^ Active (Roche Diagnostics) point of care glucometers and the Accucheck^®^ Active strips.

To address potential sources of confounding, covariates were added based on theory and their assumed effect on the latent and outcome variables. Directed acyclic graphs were used to determine if a variable should be considered as a potential confounder. Education and BMI were taken into account in the analysis of motivational constructs. Age, sex, occupation, BMI, and education were taken into account in the analysis of PA outcomes (Bauman et al., 2012).

### Contextual Adaption

All measures were translated into the local language of the study population (i.e. Lusoga) and adapted to the local context based on inputs from a local team of research assistants. Measures were then back translated to English and adjustments made where necessary to ensure that the meaning was not lost. Local validity was ensured through piloting in a non-study area, training of data collectors (e.g. through mock interviews), and minimizing inter-interviewer variability.

### Data Analysis

Data were analyzed using R software with the packages “lavaan” and “semTools.” Confirmatory factor analysis was used to test whether the item indicators of controlled and autonomous motivation, perceived competence, and perceived relatedness demonstrated adequate loadings on the latent variables. The overall fit of the measurement models was tested through the comparative fit index (CFI), Tucker-Lewis index (TLI), and root mean square error of approximation (RMSEA) and its 90% confidence interval (Bollen, 1989). Acceptable model fit was defined by: RMSEA (≤0.08), SRMR (≤0.08), CFI and TLI (≥0.95) (Hu and Bentler, 1999). Multiple indices were used because they provide different information about model fit. Reported PA frequencies were treated as ordered categorical. Latent constructs were assumed normally distributed underlying observed categorical indicators which were treated as ordered categorical. Parameter estimation was based on diagonally weighted least squares. A second order model was used for controlled motivation since it included two types of motivation: external and introjected motivation. As an estimate of internal consistency, total omega was calculated to measure the proportion of test variance due to the common factors (Revelle and Zinbarg, 2009).

Structural equation modeling was used to test if our data fit the hypothesized SDT model based on measures of fit (CFI, TLI, RMSEA, and RSMR) using the same thresholds as for the measurement models. Regressions or correlations between latent constructs and outcomes were then compared to the relationships in the hypothesized model. Mediation was based on the criteria defined by Cerin (Cerin, 2010). To account for the potential effect of covariates, multiple indicator, multiple cause models (MIMIC) were used.

To explore the link between PA outcomes and HbA1C and FPG, a separate linear regression model was used to control for the following covariates: age, sex, and BMI (Boniol et al., 2017).

## Results

The study population was characterized by a high proportion of peasant farmers (71%), female participants (66%), and participants who were married or cohabiting (71%). Almost one third (29%) did not obtain a primary degree in education. Table 1 provides more detail about the study participants, including descriptive statistics of the PA outcome scores.

**TABLE 1:** Summary statistics (*N* = 712) of characteristics of the study population and PA outcomes.

Variable	*N*	Proportion	Mean ± SD
**Demographic characteristics and diagnosis**			
Age in yrs			52.62 ± 10.16
HbA1C in%			7.83 ± 2.62
FPG in mg/dl			165.2 ± 73.94
**Sex**			
Female	471	66%	
Male	241	34%	
**Education**			
No education	204	29%	
Primary education	372	52%	
Secondary education or higher	135	19%	
**Civil status**			
Married or cohabiting	503	71%	
Other	208	29%	
**Employment**			
Peasant farmer	494	69%	
Yes (other)	47	7%	
No	170	24%	
**Diagnosis**			
Diabetes	383	54%	
Pre-diabetes	329	46%	
**Body Mass Index**			
(15–18.5)	42	6%	
(18.5–25)	303	43%	
(25–30)	227	32%	
(30–35)	94	13%	
(30–60)	40	6%	

**PA outcomes**	**Median**	**IQ-range**	**Missing (*N*)**

Vigorous PA (≥15 min.)	5	2–7	1
Moderate PA (≥30 min.)	6	3–7	2
No of steps in 7 days	34705	24180–51508	72
No of steps per day	5344	3454–7358	72

*SD = standard deviation, IQ-range = interquartile range, FPG = fasting plasma glucose, HbA1C = glycated hemoglobin, PA = physical activity.*

### Measurement Models

The measurement models contained no double-loading indicators and measurement error of indicators was presumed uncorrelated. For each group of indicators, the unstandardized parameters were all significant (*z* > 1.96) with the lowest completely standardized parameter equaling 0.591 (for controlled motivation). Measures of fit for the constructs of perceived competence, perceived relatedness, and autonomous motivation were excellent ranging from 0.99 to 1 for CFI, from 0.99 to 1 for TLI, from 0.023 to 0.056 for SRMR, and from 0.011 to 0.068 for RMSEA. For the same constructs, estimates of total omega were 0.81, 0.81, and 0.66. Estimates of unstandardized and completely standardized parameters of the full (structural) model are reported in “Supplementary File 2.”

### Structural Model

**FIGURE 2:**
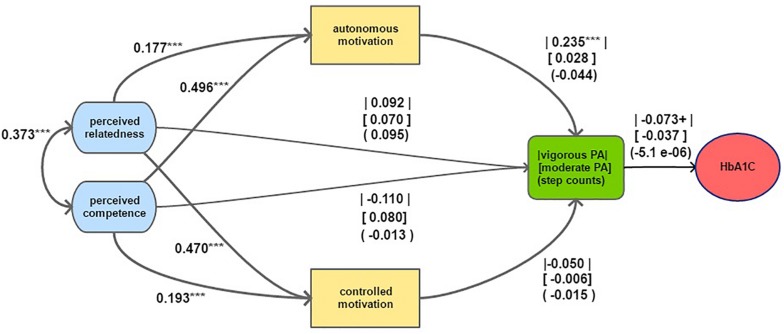
Structural model of motivational constructs predicting physical activity combined with the linear model predicting HBA1C. Note: The models control for covariates as reported in the “Materials and Methods” section. Parameter estimates are fully standardized. Parameters between straight lines, parentheses and square brackets refer to the estimates with regards to vigorous PA frequency, moderate PA frequency and pedometer counts, respectively. HbA1C was predicted in percentage points. *P*-value codes: < 0.1 ‘ + ’, *p* < 0.05 ‘*’, *p* < 0.1 ‘**’, *p* < 0.005 ‘***’.

Table 2 presents bivariate correlations between latent and outcome variables. The model was tested as it was hypothesized in the introduction for each outcome of PA. Exogenous covariates were added as described in the “Materials and Methods” section under measures. The model was identified (degrees of freedom = 294). As expected from the measurement models, factor loadings ranged from acceptable to high (all above 0.55) (see Supplementary File 2 for more detail). Fitting the proposed model to our data resulted in a good fit for the three PA outcomes. For the unadjusted model (i.e. with no covariates included): CFI ≥ 0.979, TLI ≥ 0.975, RMSEA ≤ 0.064 (90% CI ≤ 0.059–0.069), and SRMR ≤ 0.081. For the adjusted model: CFI ≥ 0.971, TLI ≥ 0.981, RMSEA ≤ 0.055 (90% CI ≤ 0.051–0.059), and SRMR ≤ 0.082. Direct effects of the fully standardized regression parameter estimates are displayed in Figure 2. Estimates of unstandardized regression parameters and their corresponding standard errors and *p*-values are reported in Supplementary File 2.

**TABLE 2:** Correlations between latent and outcome variables.

	PC	PR	AM	CM
Perceived competence (PC)	1			
perceived relatedness (PR)	0.389	1		
Autonomous motivation (AM)	0.561	0.339	1	
Controlled motivation (CM)	0.288	0.553	0.178	1
Vigorous PA frequency	0.036	0.147	0.192	0.056
Moderate PA frequency	0.136	0.144	0.113	0.081
Pedometer counts	0.011	0.086	−0.01	0.009

Combined effects (i.e. direct and indirect effects taken together) and indirect effects of the fully standardized regression parameter estimates are displayed in Table 3.

**TABLE 3:** Regression estimates of the structural equation model testing SDT and of the linear regression model testing the association between HbA1C and PA, and FPG and PA, each for different PA outcomes.

	Vigorous PA	Moderate PA	Step counts
**Combined effects**			
Perceived competence → PA outcome	0.038	0.120*	–0.008
Perceived relatedness → PA outcome	0.109**	0.107*	0.066^+^
Perceived competence → Autonomous motivation	0.562***	0.562***	0.562***
Perceived relatedness → Autonomous motivation	0.362***	0.362***	0.362***
Perceived competence → Controlled motivation	0.369***	0.369***	0.369***
PERCEIVED relatedness → Controlled motivation	0.543***	0.542***	0.542***
**Indirect effects: mediated via autonomous motivation**			
Perceived competence → PA outcome	0.131***	0.018	–0.029
Perceived relatedness → PA outcome	0.085***	0.012	–0.019

Regression estimates of the structural equation model are fully standardized. Combined effects refer to the combination of direct and indirect effects. The models control for covariates as reported in the “Materials and Methods” section. HbA1C was predicted in percentage points and FPG in mg/dl. *p*-value < 0.1 “+”, *p* < 0.05 “*”, *p* < 0.1 “**”, *p* < 0.005 “***.”

Figure 2 displays the model indicating a significant association between autonomous motivation and vigorous PA with a standardized regression coefficient of 0.24 which means that 1 unit (or 1 standard deviation) increase in autonomous motivation leads to a 0.24 unit (or 0.24 standard deviation) increase in self-reported days of vigorous PA. For moderate PA and pedometer counts, the association with autonomous motivation was not significant. None of the associations between controlled motivation and the PA outcomes were significant.

The combined effects in Table 3 indicate that perceived competence and relatedness were each strongly associated with both types of motivation. Because of the positive association between autonomous motivation and vigorous PA, this implies that autonomous motivation met the criteria of a mediator between the psychological needs and vigorous PA. In addition, the indirect effects of perceived competence and relatedness via autonomous motivation had *P*-values of 0.001 and 0.002, respectively. The indirect effects between the same constructs had *P*-values higher than 0.4 for moderate PA and for pedometer counts. The combined effect of perceived relatedness on the outcomes was significant for all types of PA, but the model indicated a weaker association for step counts. Perceived competence, however, was only associated with the moderate PA outcome and neither with vigorous PA nor with step counts.

The unadjusted linear regression model showed a positive association between HbA1C and vigorous and moderate PA (see Table 4). However, after adjusting for age, sex, and BMI, only the association with vigorous PA remained (see Table 4: linear regression models). The regression estimate of −0.073 implies that per extra day of self-reported vigorous PA per week, participants’ HbA1C decreases with 0.073 percentage points while holding age, sex, and BMI constant. The model did not show any association between HbA1C and step counts. With regards to FPG, a similar trend occurred with only vigorous PA being negatively association with FPG.

**TABLE 4:** Regression estimates of the linear regression model testing the association between HbA1C and PA, and FPG and PA, for different PA outcomes.

	Vigorous PA	Adjusted	Moderate PA	Adjusted	Step counts	Adjusted
HbA1C∼PA	−0.101**	−0.073^+^	−0.083^+^	–0.037	2.064 e-06	−5.16 e-06
*P*-value	0.007	0.054	0.053	0.397	0.680	0.314
FPG∼PA	−2.728*	−2.466*	–1.954	–1.212	2.333 e-06	−2.104 e-04
*P*-value	0.011	0.022	0.109	0.329	0.987	0.150

Adjusted models control for the covariates as reported in the “Materials and Methods” section. HbA1C is predicted in percentage points and FPG in mg/dl. *p*-value < 0.1 “+”, *p* < 0.05 “*”, *p* < 0.1 “**.”

## Discussion

This is the first published study that provides evidence for SDT in explaining regular PA in a rural sub-Saharan African population at risk of, or living with, T2D. Our data support a positive effect of autonomous motivation on PA frequency which was in turn linked to a lower HbA1C and FPG. Further in line with SDT, we found no association between controlled motivation and PA frequency. However, findings also suggested that motivation played a different role in different types of PA, which is in line with previous studies (Teixeira et al., 2012). In line with the basic psychological needs theory we found a positive effect of perceived autonomy and relatedness on autonomous motivation. Our data showed an excellent fit to the hypothesized model, which supports our findings.

The positive effect of autonomous motivation on vigorous PA frequency and the lack of association between controlled motivation and each of the PA outcomes are in line with western studies and in support of SDT (Teixeira et al., 2012). However, our data did not replicate a positive association between autonomous motivation and moderate PA frequency or pedometer counts. The lack of association with moderate PA can be explained statistically: a high proportion (47%) of the study population had a maximum score of 7, which led to a relatively small variance. The lack of association may also have been due to what our study population conceptualized as moderate PA. The concept was partly presented as mundane or repetitive work-related PA (among other items, the question referred to gardening and heavy cleaning such as washing windows, vacuuming, sweeping, or mopping). This may also explain the high score on this outcome in a study population of which the majority (69%) is working as peasant farmers in a rural African context. In a recent study in Uganda by Guwatudde et al. (2016), study participants also reported high scores of moderate PA, of which work-related activities accounted for more than 50%. If a substantial part of moderate PA indeed reflects repetitive work-related activities rather than deliberate exercising, people were likely moved by a form of external or less autonomous motivation, which was not specifically explored in this study. Because of the same idea, pedometer counts may also have depended strongly on work-related activities, including commuting by foot, which in this setting is typically not volitional. Future studies may further explore these findings through explicitly asking participants about work-related motives for PA and differentiating between work-related and exercise-related PA outcomes. The different patterns of association for different PA domains is in line with previous studies evaluating PA and SDT (Teixeira et al., 2012). In line with those studies, our findings support the idea that different subtypes of motivation explain different types of PA.

Self-reported vigorous PA was linked to a decrease in HbA1C and FPG, but we did not find this association for the other PA outcomes. Abundant evidence exists on the inverse relationship between PA and HbA1C and FPG, but most of those studies were based on an experimental design (Boniol et al., 2017). Because of the time lag between the actual behavior change and the change in biomarkers, the cross-sectional design of our study may explain the lack of association with moderate PA and pedometer counts and the relatively small effect of vigorous PA. Another reason that may explain this lack of association with moderate PA is the limited variance in this variable, as was mentioned before. Finally, it is possible that the intensity of PA (vigorous versus moderate) may have played a role in its effect on those biomarkers, in line with a recent meta-analysis which found that high-intensity exercising had a greater beneficial effect on HbA1C than low-to-moderate-intensity exercising (Liu et al., 2019).

To the best of our knowledge, this is the first study providing evidence for a positive association between autonomous motivation and perceived competence (measured through barrier self-efficacy), as well as perceived relatedness (measured through perceived participation of significant others) with regards to PA in a sub-Saharan African context. Strong conceptual parallels were found between self-efficacy and perceived competence (Mcneill et al., 2006; Fortier et al., 2007; Sweet et al., 2009) and the measure of perceived participation of significant others was used in other studies to measure the concept of perceived relatedness (Levy and Cardinal, 2004). Despite a conceptual difference with the actual measures of the basic psychological needs, we therefore conclude that our findings are in line with the basic psychological needs theory. The theory posits that satisfaction of psychological needs will nurture more autonomous forms of motivation, which will finally result in more favorable behavioral outcomes (Deci and Ryan, 2000). This implies that autonomous forms of motivation also function as a mediator between those needs and behavioral outcomes. Our data support this mediation effect of autonomous motivation for vigorous PA frequency: the indirect effect of perceived competence and relatedness on vigorous PA via autonomous motivation was positive, as well as the direct effects of these constructs (i.e. perceived competence and relatedness on autonomous motivation and autonomous motivation on vigorous PA). While the total effect of perceived competence on vigorous PA frequency was not significant, this is not a necessary condition for the occurrence of significant mediated effects between them (Cerin, 2010). A nil total effect could be explained by the effect of perceived competence triggering other mechanisms whose effects are of the opposite direction, as suggested by the negative coefficient of the direct effect of perceived competence on vigorous PA frequency (Cerin, 2010). Potential reasons for this are discussed below.

Our data confirm the positive association between perceived relatedness (measured as perceived participation of significant others: friends, family, or relatives) and PA frequency. This is in line with the association between social support and PA which was found in numerous studies in western countries (Sallis et al., 2016) and a limited number of studies in Sub-Saharan Africa (Sallis et al., 2016). Surprisingly, previous studies analyzing SDT did not find a consistent link between perceived relatedness and PA (Teixeira et al., 2012). This lack of association has been attributed to the context, the type of PA, and the measures used to assess perceived relatedness (Teixeira et al., 2012). The measure used in this study was based on a widely used and validated scale and focuses on the frequency of participation of significant others. We believe that using the frequency of participation resulted in a more objective comparison among participants, but it may have underestimated the actual support participants experienced from significant others. A sign of the latter may have been the strong association between our measure of perceived relatedness and controlled motivation which includes introjected motivation (e.g. feelings of guilt, shame, etc.) and external motivation (e.g. perceived pressure from others).

Surprisingly, the combined effect of perceived competence (measured as barrier self-efficacy) was only associated with moderate PA and not with the other PA outcomes. Barrier self-efficacy, however, has been found to be a consistent predictor of PA in western countries and, more recently, also in sub-Saharan African countries (Sallis et al., 2016). A potential explanation of the difference in significance of barrier self-efficacy among different levels of intensity of PA is participants’ understanding of the measurement items. Recent studies suggest that self-efficacy measured through “can do” questions reflects a motivational construct rather than the concept of perceived competence as proposed by SDT (Williams and Rhodes, 2016). Those studies argue that motivational mechanisms determining health behavior are not limited to the goal-directed constructs proposed by a content theory like SDT, but also include other processes: e.g. affective and non-conscious processes, perceived temporal proximity of behavioral outcomes, competing motives for alternative behaviors, etc. (Williams and Rhodes, 2016). Our data suggest that if such alternative constructs would play a role in explaining PA, they would depend on the intensity and/or type of the activity. For example, motivation to perform more challenging tasks (i.e. vigorous PA in this study) may be better explained by goal-orientation theories like SDT. In line with what we stated before, the activities conceptualized under moderated PA may have been perceived differently (e.g. less challenging), and hence moderate PA may have been better explained by those alternative types of motivation. Studies have investigated alternative psychological constructs combined with the constructs of the theory of planned behavior (Williams and Rhodes, 2016). Future research may also combine components of such alternative theories with constructs of SDT to improve the understanding of motivation in health behavior and the interaction between constructs of alternative theories and the SDT constructs.

Theoretical and health policy implications of our findings from a rural Ugandan context include that interventions aiming to increase PA will be more successful if they focus on people’s willingness to take responsibility over their health by emphasizing health-related benefits, rather than focusing on external triggers (e.g. social pressure, incentives, etc.) or introjected regulation (e.g. by blaming someone or making the individual feel guilty or ashamed). The positive effect of perceived relatedness and competence emphasizes the importance of the individual’s social environment. More specifically, people need to experience support from significant others, which implies that programs can benefit from including specific forms of social support (e.g. through group activities, peer support, family support, etc.) as a core component of their strategy to motivate individuals. However, in doing so, it is essential that individuals experience genuine support from those significant others. Secondly, it is crucial that such support contributes to positive reinforcement and to the individual’s endorsement of their own skills and competences, rather than triggering competition or social pressure among participants, which will probably not result in a higher PA frequency according to our findings. Blaming individuals or making them feel bad or guilty about a lack of engagement in PA is unlikely to contribute to their motivation or long-term performance.

### Limitations and Future Directions

The cross-sectional design of this study does not allow for evaluating changes over time. While it is important to understand motivational dynamics in recently diagnosed people, different dynamics may become relevant in people with long-standing diabetes. In addition, this design does not provide evidence for causal pathways. Long-term studies measuring change at different time points and intervention trials can address those shortcomings.

Most findings in this study correspond to findings in studies conducted in other western contexts. This study supports the claim that SDT is cross-culturally valid. However, to fully confirm this claim, we encourage multi-country studies comparing the SDT model using adequate statistical techniques (e.g. multiple-group structural equations).

This study relied for a substantial part on self-reported measures. This may have contributed to shared method variance between measures which can lead to overestimation of relationships (Doty and Glick, 1998). In addition, self-reported measures are subject to recall and social desirability bias.

In previous studies, perceived autonomy has been shown to be an important psychological need nurturing more autonomous forms of motivation. Perceived autonomy was not included in this study because of the absence of contextually adapted measures at the start of the study. We encourage future studies to focus on scale development and validation of the concept of perceived autonomy in different contexts.

## Conclusion

This is the first study in a rural sub-Saharan African population at risk of, or living with, T2D investigating the relationship between PA outcomes and SDT. The study lends support to the tenets of SDT that autonomous motivation (i.e. identified regulation) has a favorable effect on PA engagement, which was linked to a decrease in HbA1C and FPG. We found no association with controlled forms of motivation (i.e. external and introjected regulation). In line with the basic psychological needs theory, perceived relatedness and competence were positively associated with PA behavior and identified autonomous motivation functioned as a mediator between those needs and PA behavior. Major recommendations for public health interventions or counselors targeting populations in a similar context include: (1) promote PA-related health benefits that people can identify with, (2) encourage genuine social support from friends or family members, (3) stimulate positive reinforcement of people’s skills and competences, and (4) avoid utilizing perceived social pressure or feelings of guilt about a perceived lack of effort as they are not likely to contribute to PA engagement.

## Data Availability Statement

The datasets generated for this study are available on request to the corresponding author.

## Ethics Statement

The studies involving human participants were reviewed and approved by the Higher Degrees, Research and Ethics Committee (HDREC) of Makerere University School of Public Health and registered with the Uganda National Council for Science and Technology. The patients/participants provided their written informed consent to participate in this study.

## Author Contributions

PA, MD, JV, and JD played a major role in the conception of the study. MD, JV, and DG played a major role in the design of the study. DG, GN, and FK were in charge of the data acquisition. JD drafted the manuscript and analyzed and interpreted the data. PA, JV, RR, and EW contributed to the interpretation of the data. All authors critically revised the work and read and approved the final manuscript.

## Conflict of Interest

The authors declare that the research was conducted in the absence of any commercial or financial relationships that could be construed as a potential conflict of interest. The reviewer JN declared a shared affiliation, with no collaboration, with several of the authors, GN, FK, and DG, to the handling Editor at the time of review.

## Abbreviations

FPG: fasting plasma glucose
HbA1C: glycated hemoglobin
PA: physical activity
SDT: self-determination theory
T2D: type 2 diabetes.
