# Supplementary material for: What Motivates People With (Pre)Diabetes to Move? Testing Self-Determination Theory in Rural Uganda
Source: Front Psychol. 2020 Mar 24;11:404. doi: 10.3389/fpsyg.2020.00404 (PMC7105875; doi:10.3389/fpsyg.2020.00404)
Supplement: Supplementary file 1 [file Data_Sheet_1.docx]

**Supplementary File 1: Questions asked to study participants**

| BEHAVIOURAL MEASURES – PHYSICAL ACTIVITY |
| --- |
| Please think about the last ONE MONTH and the last SEVEN DAYS when you answer these questions. |
| On how many of the last SEVEN DAYS did you do **vigorous activities** for **at least 15 minutes**, such as cycling uphill or at fast pace; swimming laps; carrying heavy loads; shovelling or digging; jogging; running or a sport? |
| On how many of the last SEVEN DAYS did you do **moderate activities** for **at least 30 minutes**, such as recreational swimming; gardening; heavy cleaning such as washing windows, vacuuming, sweeping or mopping; brisk walking; biking at moderate pace; etc.? |

| PARTICIPATION OF SIGNIFICANT OTHERS |
| --- |
| Intro: **We want to understand to what extent people close to you (friends, family or relatives) have helped you to do physical activity.** |
| How often have people close to you (friends, family or relatives) **exercised with you**? |
| How often have people close to you (friends, family or relatives) **encouraged you to exercise**? |
| How often have people close to you (friends, family or relatives) **changed their schedule so you could exercise together**? |
| How often have people close to you (friends, family or relatives) **discussed exercising with you**? |
| How often have people close to you (friends, family or relatives) **helped you with exercising on special occasions such as holidays, feasts, family gatherings**? |
| Answer options:  1= Never  2= less than once a week  3= once a week  4= more than once a week  888= no answer/not applicable |

| SELF-EFFICACY |
| --- |
| Intro: **We want to know if you can do physical activity under specific circumstances.** |
| Do you think you can be physically active **even during holidays, weddings or other special events**? |
| Do you think you can be physically active **even if your family does not encourage you to be physically activity**? |
| Do you think you can be physically active **even if you think it is not the best weather for doing sports**? |
| Do you think you can be physically active **even if you are in a place with no exercise facilities or safe roads for walking**?  (probing ‘for instance at home or at work or in another way’) |
| Do you think you can be physically active **even if you have health problems?** |
| Do you think you can be physically active **even if no other people around you are doing exercise or walking**? |
| Answer options:  1 Strongly disagree  2 Disagree  3 Neutral  4 Agree  5 Strongly agree  888 Not applicable |

| AUTONOMOUS AND CONTROLLED MOTIVATION |
| --- |
| Intro: Think for a moment about the **reasons** why you actually would do physical exercise: why would you do this?  (*Instructions: give the patient a couple of seconds to think about it*).  We will now present possible reasons why you may do physical exercise. Please agree or disagree, using the options provided. |
| Would you do physical exercise **because** **you personally believe it is the best thing for your health**? |
| Would you do physical exercise **because** **you'd feel bad about yourself if you didn't**? |
| Would you do physical exercise **because** **you feel pressure from others to do it**? |
| Would you do physical exercise **because** **others would be upset with you if you didn't**? |
| Would you do physical exercise **because it is very important for being as healthy as possible**? |
| Would you do physical exercise **because** **you would feel guilty or ashamed of yourself if you didn’t**? |
| Would you do physical exercise **because you feel that you want to take responsibility for your own health**? |
| Would you do physical exercise **because** **it is an important choice you really want to make**? |
| Answer options:  1 Strongly disagree  2 Disagree  3 Neutral  4 Agree  5 Strongly agree  888 Not applicable |
