# Supplementary material for: What Motivates People With (Pre)Diabetes to Move? Testing Self-Determination Theory in Rural Uganda
Source: Front Psychol. 2020 Mar 24;11:404. doi: 10.3389/fpsyg.2020.00404 (PMC7105875; doi:10.3389/fpsyg.2020.00404)
Supplement: Supplementary file 2 [file Data_Sheet_2.docx]

# Supplementary File 2: Estimates of factor loadings and regression parameters

Factor loadings are reported under “Latent variables”, single effects are reported under “Regressions” and total effects (direct and indirect combined) are reported under “Defined Parameters”. In the first five columns: unstandardized parameters (estimate) with their standard errors (std.Err), related t-values and p-values. In the last two columns: the parameters with only the latent variable standardized (Std.lv) and completely standardized (Std.all) parameters. PR = perceived relatedness, PC= perceived competence, AM = autonomous motivation, CM = controlled motivation, pa15 = vigorous PA frequency, pa30= moderate PA frequency, pedct= pedometer counts, BMI = BMI, sexf = sex, educf = levels of education, emplf = employment status.

1. **Outcome: vigorous PA frequency**

Latent Variables:

Estimate Std.Err t-value df P(>|t|) Std.lv Std.all

PR =~

ssa1 1.000 0.885 0.885

ssa2 0.992 0.021 48.033 Inf 0.000 0.878 0.878

ssa3 1.043 0.019 55.112 Inf 0.000 0.923 0.923

ssa4 0.981 0.020 49.555 Inf 0.000 0.869 0.869

ssa5 0.904 0.028 32.456 Inf 0.000 0.801 0.801

PC =~

effb7 1.000 0.697 0.697

effb8 1.047 0.055 19.077 Inf 0.000 0.730 0.730

effb9 1.136 0.057 19.851 Inf 0.000 0.792 0.792

effb10 1.001 0.056 17.908 Inf 0.000 0.698 0.698

effb11 0.796 0.059 13.483 Inf 0.000 0.555 0.555

effb12 0.898 0.056 15.901 Inf 0.000 0.626 0.626

AM =~

srb1 1.000 0.824 0.819

srb5 0.911 0.051 17.839 Inf 0.000 0.751 0.747

srb7 0.961 0.054 17.887 Inf 0.000 0.792 0.788

srb8 0.872 0.054 16.244 Inf 0.000 0.718 0.715

CM1 =~

srb2 1.000 0.833 0.831

srb6 0.997 0.044 22.839 Inf 0.000 0.831 0.829

CM2 =~

srb3 1.000 0.574 0.574

srb4 1.800 0.171 10.551 Inf 0.000 1.033 1.032

CM =~

CM1 1.000 0.981 0.981

CM2 0.523 0.064 8.107 Inf 0.000 0.744 0.744

Regressions:

Estimate Std.Err t-value df P(>|t|) Std.lv Std.all

AM ~

PC (a) 0.586 0.063 9.329 Inf 0.000 0.496 0.496

PR (b) 0.164 0.050 3.270 Inf 0.001 0.177 0.177

CM ~

PC (c) 0.226 0.052 4.383 Inf 0.000 0.193 0.193

PR (d) 0.434 0.041 10.658 Inf 0.000 0.470 0.470

pa15 ~

AM (e) 0.302 0.083 3.657 Inf 0.000 0.249 0.235

CM (f) -0.065 0.078 -0.835 Inf 0.404 -0.053 -0.050

PC (g) -0.167 0.088 -1.891 Inf 0.059 -0.116 -0.110

PR (h) 0.110 0.070 1.571 Inf 0.116 0.098 0.092

Sexf 0.341 0.093 3.645 Inf 0.000 0.341 0.152

age -0.008 0.004 -2.059 Inf 0.039 -0.008 -0.082

educf_2 0.188 0.103 1.821 Inf 0.069 0.188 0.089

educf_3 0.388 0.138 2.821 Inf 0.005 0.388 0.144

emplf_1 0.644 0.169 3.817 Inf 0.000 0.644 0.281

emplf_2 0.111 0.179 0.621 Inf 0.534 0.111 0.045

BMI -0.015 0.007 -2.123 Inf 0.034 -0.015 -0.078

AM ~

educf_2 0.242 0.099 2.449 Inf 0.014 0.293 0.147

educf_3 0.185 0.124 1.488 Inf 0.137 0.225 0.088

CM ~

educf_2 -0.065 0.087 -0.743 Inf 0.457 -0.080 -0.040

educf_3 -0.017 0.122 -0.142 Inf 0.887 -0.021 -0.008

BMI 0.009 0.007 1.372 Inf 0.170 0.011 0.063

AM ~

BMI -0.004 0.008 -0.503 Inf 0.615 -0.005 -0.026

Covariances:

Estimate Std.Err t-value df P(>|t|) Std.lv Std.all

PR ~~

PC (i) 0.230 0.028 8.236 Inf 0.000 0.373 0.373

Defined Parameters:

Estimate Std.Err t-value df P(>|t|) Std.lv Std.all

TotalPConpa15 0.026 0.063 0.406 Inf 0.685 0.040 0.038

totalPRonpa15 0.131 0.048 2.712 Inf 0.007 0.115 0.109

totalPConAM 0.624 0.057 10.986 Inf 0.000 0.562 0.562

totalPRonAM 0.299 0.046 6.544 Inf 0.000 0.362 0.362

totalPConCM 0.326 0.048 6.750 Inf 0.000 0.369 0.369

totalPRonCM 0.486 0.038 12.793 Inf 0.000 0.543 0.543

indirectPConpa15 0.187 0.055 3.422 Inf 0.001 0.139 0.131

indirectPRonpa15 0.090 0.029 3.124 Inf 0.002 0.090 0.085

1. **Outcome: moderate PA frequency**

Latent Variables:

Estimate Std.Err t-value df P(>|t|) Std.lv Std.all

PR =~

ssa1 1.000 0.885 0.885

ssa2 0.992 0.021 47.936 Inf 0.000 0.878 0.878

ssa3 1.043 0.019 54.993 Inf 0.000 0.923 0.923

ssa4 0.981 0.020 49.361 Inf 0.000 0.868 0.868

ssa5 0.905 0.028 32.438 Inf 0.000 0.801 0.801

PC =~

effb7 1.000 0.698 0.698

effb8 1.045 0.055 19.113 Inf 0.000 0.729 0.729

effb9 1.134 0.057 19.837 Inf 0.000 0.792 0.792

effb10 0.999 0.056 17.882 Inf 0.000 0.698 0.698

effb11 0.796 0.059 13.501 Inf 0.000 0.556 0.556

effb12 0.898 0.056 15.947 Inf 0.000 0.627 0.627

AM =~

srb1 1.000 0.824 0.819

srb5 0.911 0.051 17.896 Inf 0.000 0.751 0.747

srb7 0.963 0.053 18.001 Inf 0.000 0.793 0.789

srb8 0.871 0.053 16.288 Inf 0.000 0.717 0.714

CM1 =~

srb2 1.000 0.834 0.832

srb6 0.995 0.044 22.846 Inf 0.000 0.830 0.828

CM2 =~

srb3 1.000 0.574 0.573

srb4 1.802 0.171 10.531 Inf 0.000 1.034 1.032

CM =~

CM1 1.000 0.981 0.981

CM2 0.521 0.064 8.107 Inf 0.000 0.743 0.743

Regressions:

Estimate Std.Err t-value df P(>|t|) Std.lv Std.all

AM ~

PC (a) 0.585 0.063 9.345 Inf 0.000 0.496 0.496

PR (b) 0.164 0.050 3.271 Inf 0.001 0.177 0.177

CM ~

PC (c) 0.227 0.052 4.387 Inf 0.000 0.194 0.194

PR (d) 0.435 0.041 10.668 Inf 0.000 0.470 0.470

pa30 ~

AM (e) 0.036 0.086 0.417 Inf 0.676 0.030 0.028

CM (f) -0.008 0.080 -0.098 Inf 0.922 -0.006 -0.006

PC (g) 0.120 0.097 1.230 Inf 0.219 0.084 0.080

PR (h) 0.083 0.071 1.178 Inf 0.239 0.074 0.070

sexf 0.502 0.097 5.158 Inf 0.000 0.502 0.227

age -0.010 0.004 -2.448 Inf 0.014 -0.010 -0.102

educf_2 0.119 0.111 1.069 Inf 0.285 0.119 0.057

educf_3 0.131 0.147 0.891 Inf 0.373 0.131 0.049

emplf_1 0.532 0.160 3.332 Inf 0.001 0.532 0.234

emplf_2 0.412 0.175 2.355 Inf 0.019 0.412 0.167

BMI -0.009 0.008 -1.171 Inf 0.241 -0.009 -0.047

AM ~

educf_2 0.242 0.099 2.449 Inf 0.014 0.293 0.147

educf_3 0.185 0.124 1.487 Inf 0.137 0.225 0.088

CM ~

educf_2 -0.065 0.088 -0.742 Inf 0.458 -0.080 -0.040

educf_3 -0.017 0.122 -0.142 Inf 0.887 -0.021 -0.008

BMI 0.009 0.007 1.373 Inf 0.170 0.011 0.063

AM ~

BMI -0.004 0.008 -0.502 Inf 0.616 -0.005 -0.026

Covariances:

Estimate Std.Err t-value df P(>|t|) Std.lv Std.all

PR ~~

PC (i) 0.231 0.028 8.236 Inf 0.000 0.373 0.373

Defined Parameters:

Estimate Std.Err t-value df P(>|t|) Std.lv Std.all

totalPConpa30 0.159 0.067 2.371 Inf 0.018 0.126 0.120

totalPRonpa30 0.118 0.050 2.350 Inf 0.019 0.112 0.107

totalPConAM 0.623 0.057 11.015 Inf 0.000 0.562 0.562

totalPRonAM 0.299 0.046 6.544 Inf 0.000 0.362 0.362

totalPConCM 0.327 0.048 6.762 Inf 0.000 0.369 0.369

totalPRonCM 0.487 0.038 12.811 Inf 0.000 0.542 0.542

indirectPConpa30 0.025 0.053 0.472 Inf 0.637 0.019 0.018

indirectPRonpa30 0.012 0.026 0.467 Inf 0.641 0.012 0.012

1. **Outcome: pedometer counts**

Latent Variables:

Estimate Std.Err t-value df P(>|t|) Std.lv Std.all

PR =~

ssa1 1.000 0.885 0.885

ssa2 0.992 0.021 47.785 Inf 0.000 0.878 0.878

ssa3 1.042 0.019 54.616 Inf 0.000 0.923 0.923

ssa4 0.982 0.020 49.134 Inf 0.000 0.869 0.869

ssa5 0.905 0.028 32.282 Inf 0.000 0.801 0.801

PC =~

effb7 1.000 0.698 0.698

effb8 1.046 0.055 18.944 Inf 0.000 0.730 0.730

effb9 1.135 0.058 19.729 Inf 0.000 0.793 0.793

effb10 1.001 0.056 17.784 Inf 0.000 0.699 0.699

effb11 0.794 0.059 13.370 Inf 0.000 0.554 0.554

effb12 0.896 0.057 15.784 Inf 0.000 0.626 0.626

AM =~

srb1 1.000 0.824 0.819

srb5 0.913 0.051 17.791 Inf 0.000 0.752 0.748

srb7 0.962 0.054 17.914 Inf 0.000 0.792 0.788

srb8 0.871 0.054 16.189 Inf 0.000 0.717 0.715

CM1 =~

srb2 1.000 0.833 0.831

srb6 0.997 0.044 22.633 Inf 0.000 0.831 0.829

CM2 =~

srb3 1.000 0.574 0.574

srb4 1.799 0.172 10.466 Inf 0.000 1.033 1.031

CM =~

CM1 1.000 0.981 0.981

CM2 0.522 0.065 8.038 Inf 0.000 0.744 0.744

Regressions:

Estimate Std.Err t-value df P(>|t|) Std.lv Std.all

AM ~

PC (a) 0.586 0.063 9.269 Inf 0.000 0.496 0.496

PR (b) 0.164 0.051 3.240 Inf 0.001 0.177 0.177

CM ~

PC (c) 0.226 0.052 4.347 Inf 0.000 0.193 0.193

PR (d) 0.434 0.041 10.568 Inf 0.000 0.470 0.470

pedCt ~

AM (e) -0.053 0.076 -0.700 266.766 0.484 -0.044 -0.044

CM (f) -0.018 0.068 -0.267 1085.093 0.790 -0.015 -0.015

PC (g) -0.019 0.078 -0.240 484.413 0.810 -0.013 -0.013

PR (h) 0.107 0.063 1.699 1590.918 0.090 0.095 0.095

sexf -0.430 0.082 -5.257 1851.598 0.000 -0.430 -0.204

age -0.015 0.004 -4.203 2949.808 0.000 -0.015 -0.158

educf_2 0.211 0.094 2.237 3137.276 0.025 0.211 0.105

educf_3 0.080 0.120 0.663 2265.584 0.507 0.080 0.031

emplf_1 -0.180 0.155 -1.165 2463.314 0.244 -0.180 -0.083

emplf_2 -0.057 0.166 -0.342 1843.433 0.732 -0.057 -0.024

BMI -0.017 0.008 -2.314 1684.181 0.021 -0.017 -0.096

AM ~

educf_2 0.242 0.100 2.428 Inf 0.015 0.293 0.147

educf_3 0.185 0.125 1.474 Inf 0.140 0.224 0.088

CM ~

educf_2 -0.065 0.088 -0.736 Inf 0.462 -0.080 -0.040

educf_3 -0.017 0.123 -0.141 Inf 0.888 -0.021 -0.008

BMI 0.009 0.007 1.361 Inf 0.174 0.011 0.063

AM ~

BMI -0.004 0.008 -0.498 Inf 0.618 -0.005 -0.026

Covariances:

Estimate Std.Err t-value df P(>|t|) Std.lv Std.all

PR ~~

PC (i) 0.231 0.028 8.166 Inf 0.000 0.373 0.373

Defined Parameters:

Estimate Std.Err t-value df P(>|t|) Std.lv Std.all

totalPConpedct -0.033 0.058 -0.573 2726.828 0.567 -0.008 -0.008

totalPRonpedct 0.078 0.044 1.758 2124.280 0.079 0.066 0.066

totalPConAM 0.623 0.057 10.921 Inf 0.000 0.562 0.562

totalPRonAM 0.299 0.046 6.484 Inf 0.000 0.362 0.362

totalPConCM 0.327 0.049 6.698 Inf 0.000 0.369 0.369

totalPRonCM 0.486 0.038 12.687 Inf 0.000 0.542 0.542

indirectPConPA -0.039 0.047 -0.842 531.129 0.400 -0.029 -0.029

indirectPRonPA -0.019 0.023 -0.836 537.737 0.403 -0.019 -0.019
